# The Radiative Efficiency and Global Warming Potential of HCFC‐132b

**DOI:** 10.1002/cphc.202400632

**Published:** 2024-12-04

**Authors:** Daniela Alvarado‐Jiménez, Andrea Pietropolli Charmet, Paolo Stoppa, Nicola Tasinato

**Affiliations:** ^1^ IUSS Pavia; ^2^ Scuola Normale Superiore; ^3^ Dipartimento di Scienze Molecolari e Nanosistemi Università Ca' Foscari Venezia

**Keywords:** Climate metrics, Computational chemistry, Infrared spectroscopy, Greenhouse gases, Halocarbons

## Abstract

Hydro‐chloro‐fluoro‐carbons (HCFCs) are potent greenhouse gases which strongly absorb the infrared (IR) radiation within the 8–12 *μ*m atmospheric windows. Despite international policies schedule their phasing out by 2020 for developed countries and 2030 globally, HCFC‐132b (CH_2_ClCClF_2_) has been recently detected with significant atmospheric concentration. In this scenario, detailed climate metrics are of paramount importance for understanding the capacity of anthropogenic pollutants to contribute to global warming. In this work, the radiative efficiency (RE) of HCFC‐132b is experimentally measured for the first time and used to determine its global warming potential (GWP) over 20‐, 100‐ and 500‐year time horizon. Vibrational‐ and rotational‐spectroscopic properties of this molecule are first characterized by exploiting a synergism between Fourier‐transform IR (FTIR) spectroscopy experiments and quantum chemical calculations. Equilibrium geometry, rotational parameters and vibrational properties predicted theoretically beyond the double‐harmonic approximation are employed to assist the vibrational assignment of the experimental trace. Finally, FTIR spectra measured over a range of pressures are used to determine the HCFC‐132b absorption cross section spectrum from 150 to 3000 cm^−1^, from which istantaneous and effective REs are derived and, in turn, used for GWP evaluation.

## Graphical abstract

HCFCs are strong greenhouse gases absorbing infrared radiation. Despite plans to phase them out by 2030, HCFC‐132b was recently detected in the atmosphere. Its vibrational‐ and rotational‐spectroscopic properties, radiative efficiency, and global warming potential are measured by coupling Fourier‐transform infrared spectroscopy and quantum chemical calculations.

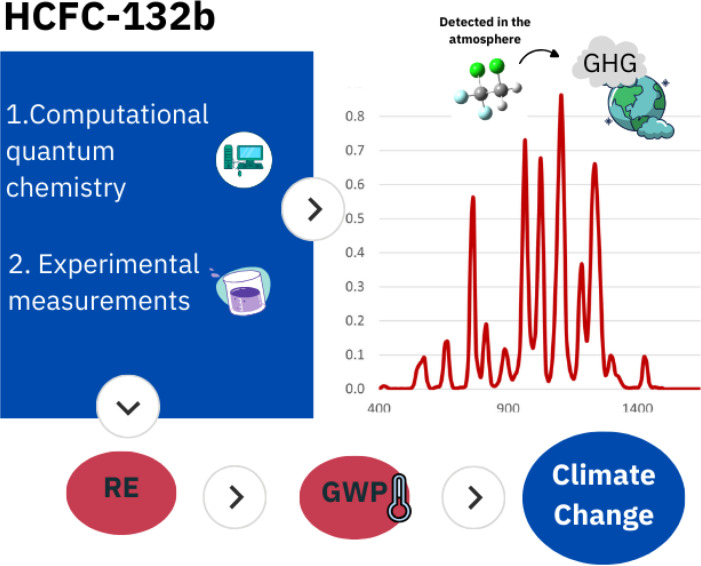

## Introduction

A variety of chemical pollutants is released into the atmosphere in large amount as a result of human activities. Once emitted, these substances are dynamically transported over the different layers of the atmosphere where they undergo oxidative chemical transformation, over a range of spatial and time scales depending on the degradation mechanism responsible for their removal which, in turn, dictates their atmospheric residence time. Either the pristine compound and the degradation products then exert a negative effect on the environment. Among anthropogenic emissions into the atmosphere, increasing levels of carbon dioxide are the foremost cause of global warming because of its indisputable role in unbalancing the Earth's radiative budget. In this respect, the concept of radiative forcing (RF) has been widely used as a key metric to measure the externally induced net energy imbalance at the top of the atmosphere capable of causing climate parameter changes and ultimately of leading a new equilibrium state.^[1]^ Based on this definition, in conjunction with atmospheric lifetimes, a molecule's radiative efficiency (RE) is established, representing the RF per unit change in a gas atmospheric concentration, thus providing a measure of its greenhouse strength. In addition, the global warming potential (GWP) index, i. e. ratio of the time‐integrated radiative efficiency from the instantaneous release of 1 kg of a trace substance relative to that of 1 kg of a reference gas, provides a standardized metric (on a CO_2_‐equivalent scale), for comparing the greenhouse capacity of non‐CO_2_ gases.

While CO_2_ is the main driver of climate change, the RF due to halogenated compounds is estimated to be about 20–25 % that of carbon dioxide.^[2]^ These compounds bear many diverse applications, being used as refrigerants, fire extinguishers, blowing agents, anesthetics, and reactants for the production of fine chemicals. In particular, chlorofluorocarbons (CFCs) have found widespread application thanks to their thermal and chemical stability that, on the other hand, make these molecules to be characterized by very long atmospheric lifetimes of hundreds or even thousands of years. As a consequence, CFCs are capable of diffusing from the emission point in the troposphere to the stratosphere where, UV irradiation from the sun, causes homolytic dissociation of the C−Cl bond with the release of chlorine atoms that triggers the catalytic destruction of stratospheric ozone, a process that has led to the appearance of the ozone hole. As a consequence, the production and use of CFCs have been banned by the Montreal Protocol on ozone‐depleting substances (ODS) and its subsequent amendments that also scheduled the phasing out for hydro‐chloro‐fluorocarbons (HCFCs) emissions by 2020 for developed countries and 2030 globally. These indeed have been considered as *ad interim* replacements of CFCs, in view of their shorter lifetimes due to the presence of at least one C−H bond that is prone to attack by the OH radical in the troposphere. Despite the existing international regulations, emissions of some ODS have been surprisingly observed to be diminishing very slowly or even increasing. In particular, a few years ago long‐term emissions in the atmosphere of three ODS, CH_2_ClCClF_2_ (HCFC‐132b), CH_2_ClCF_3_ (HCFC‐133a) and CH_2_ClF (HCFC‐31), were reported.^[3]^ Among these, HCFC‐132b has been detected with an increasing mixing ratio over the period from 2016 to 2019, with a maximum concentration of 0.17 ppt retrieved toward the end of 2019 in the northern hemisphere. This HCFC has not reported end‐uses, even though it is likely formed during the synthesis of CF_3_CF_2_H (HFC‐134a) and possibly other hydro‐fluoro‐carbons used in refrigerating systems. For this reason, it is barely regulated by the Montreal protocol that focuses on ODS with end‐use applications while posing less control on feed‐stocks and process emissions. Even though HCFC‐132b could be used as refrigerant, a few studies have dealt with this molecule, mainly because of the lack of end‐uses. Some investigations on properties like toxicity,^[4]^ compressibility,^[5]^ conductance,^[6]^ and thermodynamic functions^[7]^ have been carried out over the years, while photochemical processes^[8]^ and the kinetics of the reaction toward the OH radical,[^9^, ^10^] which are of interest from the point of view of atmospheric chemistry, have been addressed to some extent. Very recently, the mechanistic and kinetic details of the reaction between HCFC‐132b against the OH radical and the Cl atom have been analyzed by using *ab initio* triple‐slash dual‐level direct dynamics, showing that the room‐temperature H‐abstraction by the atom Cl is about 3.6 times faster than the OH‐initiated reaction.^[11]^

The vibrational and rotational spectra of HCFC‐132b have, to the best of our knowledge, never been characterized. An accurate knowledge of the vibrational‐rotational spectroscopic properties of molecules with atmospheric relevance is, on the one side, a fundamental prerequisite to exploit spectroscopic remote sensing retrievals, and, on the other, the knowledge of the absorption cross‐section infrared (IR) spectrum is necessary to measure the radiative efficiency which, in turn, coupled with the molecule atmospheric lifetime allow its GWP to be derived.[^12^, ^13^, ^14^] While the RE and GWP of HCFC‐132b have recently been reported in the last World Meteorological Organization (WMO) ozone assessment report to be 0.214 Wm^−2^ ppbv^−1^ and 332^[15]^ for a time horizon of 100 years, it has to be noted that these values do not rely on IR absorption cross section spectra measured experimentally, but rather simulated theoretically within the double‐harmonic approximation. The latter, however, is well‐known to be impacted by several drawbacks, the most important one being a general overestimation of transition frequencies, and the missing contributions stemming from overtones and combination bands. Based on the available literature on the subject,[^16^, ^17^, ^18^, ^19^] frequency overestimation ranges from a few cm^−1^ to hundreds cm^−1^ while the error in the computed intensities is, on average, twice that stemming from full anharmonic computations. While the application of empirical scaling factors can alleviate the problem of frequency overestimation, it cannot recover for the missing transitions nor introduce anharmonic effects into the computed transition strengths. Under this point of view, it has been reported that for hydrofluorocarbons the error in the predicted band strengths range from 5 to 20 % depending on the molecule.^[20]^

**Figure 1:**
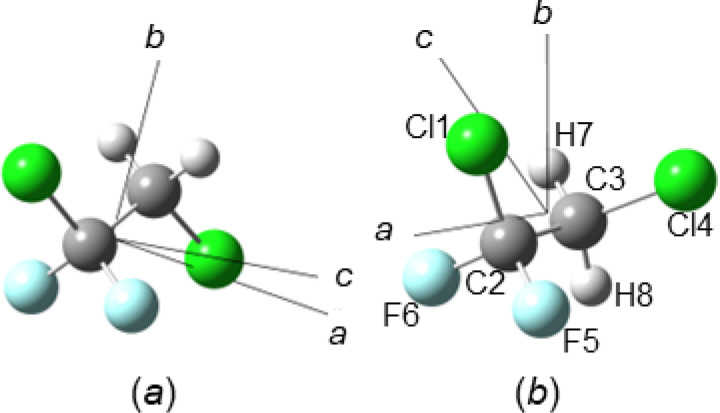
Structure of HCFC‐132b (a) *C_S_* and (b) *C*_1_ conformers with atom labelling. The orientation of the principal axes of inertia is also shown.

With these premises, the present work deals with a combined theoretical and experimental determination of the rotational and vibrational spectroscopic properties of HCFC‐132b with the final aim of deriving, for the first time, its RE and GWP. Specifically, the molecular structure and the spectroscopic parameters relevant to rotational spectroscopy are first derived by using composite schemes which are also adopted to derived IR transition frequencies and intensities beyond the double harmonic approximation. These are then used to interpret the pattern of fundamental transition frequencies of this molecule experimentally measured by medium resolution Fourier Transform Infra‐Red (FTIR) spectroscopy. It can be anticipated, at this stage, that the unexpected discrepancies initially observed between the experimentally measured and quantum chemically simulated band‐shapes have led to consideration of both conformers of this molecule, namely the *C_S_* and *C*_1_ configurations reported in Figure 1; actually, the latter one exists in two energetically and spectroscopically equivalent rotamers that can be obtained upon rotation about the C−C bond. Accounting for the relative population of the conformers, as obtained from quantum chemical calculations, to Boltzmann average the corresponding synthetic spectra has led to a one‐to‐one correspondence with the experimental observations. After interpretation of the fundamental modes of vibration, the experimental absorption cross section spectrum is measured for the first time over the spectral range 150–3500 cm^−1^ and used to derive the effective radiative efficiency accounting for both stratospheric temperature and lifetime corrections. Finally, the GWP of HCFC‐132b is derived over 20‐, 100‐ and 500‐years time horizons.

## Results and Discussion

As pointed out in the Introduction, initially only the *C_S_* conformer of the molecule has been considered for the interpretation of the IR spectrum in the fundamental band regions. However, comparison between experimental and simulated traces has soon evidenced a significantly larger number of strong absorptions in the former. Despite these, in a first instance, could be attributed to hot bands arising from the lowest energy levels, whose population can reach 60 % of the ground vibrational state, the corresponding transition frequencies were not compatible with those predicted theoretically. Hence, the second conformer, belonging to the *C*_1_ symmetry point group, has been considered which, according to DSDPBEP86/jun‐cc‐pV(T+*d*)Z calculations, is only 0.4 kcal mol^−1^ higher in energy than the *C_S_* structure, and therefore it can produce easily detectable spectral features. In the next subsection, the equilibrium geometry and the rotational spectra of the main isotopologues of both conformers are characterized theoretically. While this data is not used to in the evaluation of climate metrics, we deemed its inclusion relevant to provide an overall characterization of the rotational‐vibrational spectroscopic properties of the molecule that, to the best of our knowledge, is lacking. Subsequently, the attention is devoted to the investigation of the fundamental vibrational properties and the determination of RE and GWP, which are undertaken through an interplay between theory and experiment. Finally, the absorption cross section spectrum is measured, and used to derive experimental REs and GWPs of HCFC‐132b for the first time.

### Equilibrium Geometry and Rotational Spectroscopic Parameters

The equilibrium geometries of HCFC‐132b conformers obtained within the ChS are reported in Table 1 with atom labelling being detailed in Figure 1. As it can be seen, a part from the Cl4C3C2Cl1 dihedral angle, the most important structural variations between the *C_S_* and the *C*_1_ conformers are noted for the Cl4C3C2 and F6C2C3 valence angles, that change by about 4° in opposite directions, and the C2Cl1 and C2F6 distances that, respectively, shortens and elongates by about 0.01 Å. In passing, it is also worth noticing the reliability of the structural parameters obtained at the DSDPBEP86‐D3/jun‐cc‐pV(T+*d*)Z level of theory, which predicts valence and dihedral angles in very good agreement with ChS ones (the maximum difference being 0.3° for the Cl1C2C3C4 dihedral of the *C*_1_ conformer), and the maximum deviation in bond lengths is related to the overestimation of the C−Cl bond length, as expected from previous investigations and that, anyway, can be easily corrected by resorting to the Nano‐LEGO tool.[^21^, ^22^]

**Table 1:** Equilibrium geometry of HCFC‐132b conformers.^[*a*]^

parameter	Conf. 1 ChS^[*b*]^	Conf. 1 DSD^[*c*]^	Conf. 2 ChS^[*b*]^	Conf. 2 DSD^[*c*]^
*r*(C2‐Cl1)	1.7705	1.7786	1.7586	1.7655
*r*(C2‐C3)	1.5116	1.5156	1.5136	1.5176
*r*(C3‐Cl4)	1.7678	1.7720	1.7618	1.7655
*r*(C2‐F5)	1.3354	1.3397	1.3363	1.3406
*r*(C2‐F6)	1.3354	1.3397	1.3450	1.3500
*r*(C3‐H7)	1.0838	1.0865	1.0839	1.0866
*r*(C3‐H8)	1.0838	1.0865	1.0857	1.0886
*α*(C3C2Cl1)	108.84	108.77	113.06	113.07
*α*(C2C3Cl4)	110.97	110.97	112.22	112.26
*α*(C3C2F5)	111.47	111.58	111.10	111.20
*α*(C3C2F6)	111.47	111.58	107.51	107.57
*α*(C2C3H7)	108.88	108.92	108.89	108.92
*α*(C2C3H8)	108.88	108.92	107.45	107.46
*δ*(Cl4C3C2Cl1)	180.00	180.00	62.46	62.80
*δ*(F5C2C3Cl1)	−119.83	−119.79	−122.96	−122.99
*δ*(F6C2C3Cl1)	119.83	119.79	119.93	119.87
*δ*(H7C3C2Cl4)	−119.78	−119.79	−120.91	−120.97
*δ*(H7C3C2Cl4)	119.78	119.79	119.46	119.46

^[*a*]^ Bond lengths in Å, bond angles in deg. ^[*b*]^ Values at ChS level. ^[*c*]^ Values at DSDPBEP86‐D3/jun‐cc‐pV(T+*d*)Z level.

**Figure 2:**
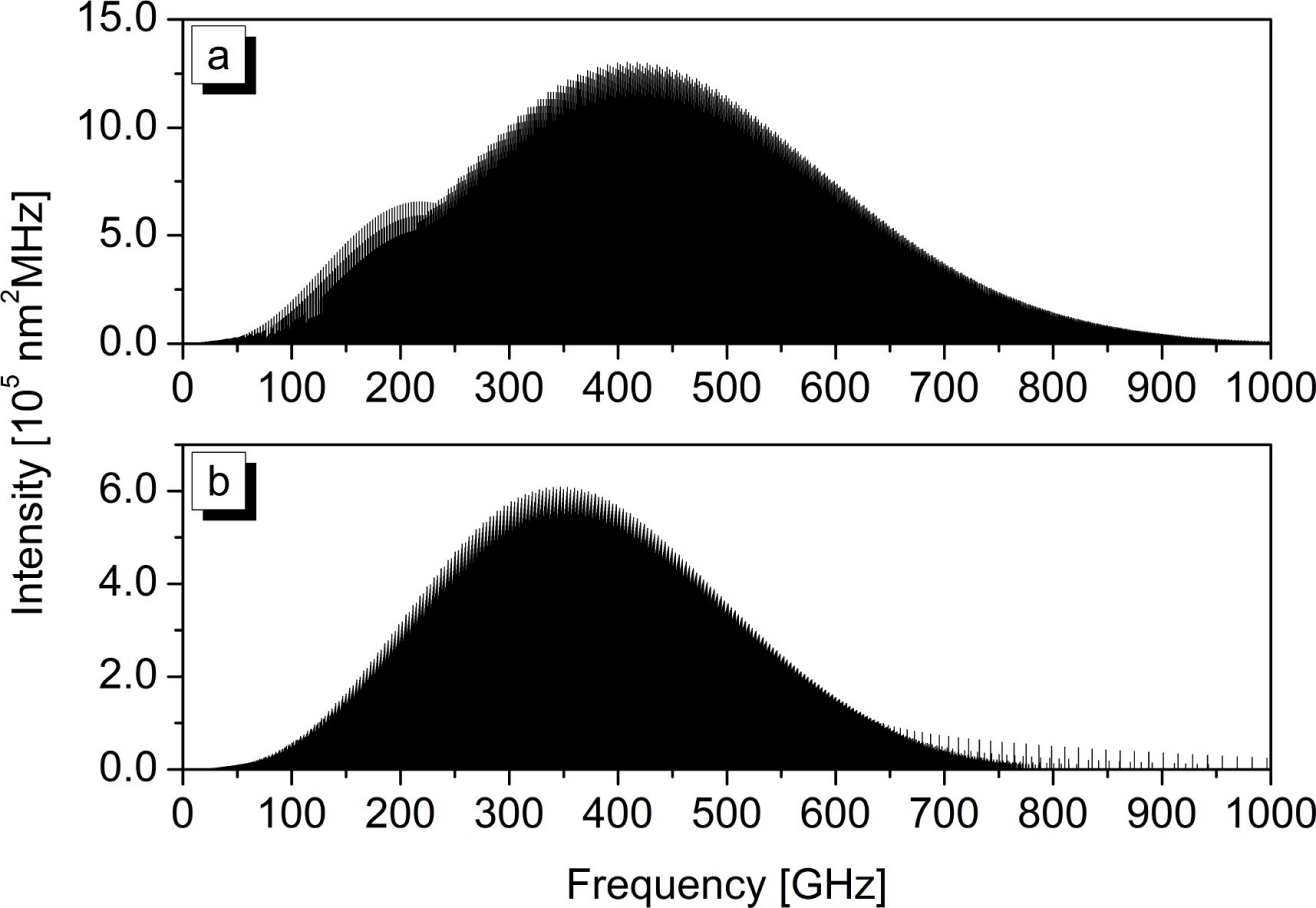
Simulated rotational spectra of HCFC‐132b between 0 and 1000 GHZ for (a) the *C_S_* and (b) the *C*_1_ rotamers.

Moving from ChS equilibrium geometries, the corresponding equilibrium rotational constants, $B_{e}^{\alpha }$ , (*α*=*a*, *b*, *c* being the principal axis of inertia) can be straightforwardly derived, from which ground state rotational constants, $B_{0}^{\alpha }$ , that can be used to drive laboratory investigations, have been obtained by correcting them through vibrational corrections perturbatively computed at the DSDPBEP86‐D3/jun‐cc‐pV(T+*d*)Z level of theory. These are reported in Table 2 together with quartic‐ and sextic‐centrifugal distortion constants, within the *A*‐ reduced Watson's Hamiltonian in the *III* ^*r*^ representation obtained again from DSDPBEP86 computations. The accuracy of the predicted rotational parameters is expected to be around 0.1 % for rotational constants[^12^, ^23^] and within 10 % for the centrifugal distortion parameters.[^17^, ^18^, ^24^] Table 2 also reports dipole moment components along the molecular principal inertial axis for both the *C_S_* and *C*_1_ conformers, which rule the intensities of their pure rotational transitions. By using the VMS‐ROT software^[25]^ and the rotational spectroscopic parameters collected in Table 2, the room temperature pure rotational spectra of both HCFC‐132b conformers have been simulated as reported in Figure 2. As it can be seen, the rotational spectra of the *C*_1_ rotamer are predicted weaker than the *C_S_* rotamer by about a factor of 2, which should be further scaled in view of the relative population of the two species. Furthermore, as the molecule contains two chlorine atoms with nuclear spin quantum number *I*=3/2, rotational transitions are expected to be split into hyperfine components. For the purpose, nuclear quadrupolar coupling constants for both Cl atoms are also listed in Table 2 thus providing a complete characterization of the rotational spectra of ^35^Cl isotopologues of HCFC‐132b rotamers which can be used to support their experimental characterization.

**Table 2:** Rotational spectroscopic parameters of HCFC‐132b rotamers of the *A*‐ reduced Watson's Hamiltonian in the *III* ^*r*^ representation.^[*a*]^

	Conf. 1	Conf. 2
*A_e_*	4647.374	3248.880
*B_e_*	1336.504	1665.238
*C_e_*	1278.266	1391.259
*A*_0_	4620.932	3232.212
*B*_0_	1328.680	1655.404
*C*_0_	1270.771	1382.895
Δ_J_	0.0966	0.2600
Δ_JK_	1.7697	−0.7706
Δ_K_	0.9061	1.1952
*δ*_J_	0.0021	0.0541
*δ*_K_	−0.0367	0.6410
Φ_J_	4.70×10^−6^	−8.09×10^−5^
Φ_JK_	3.85×10^−5^	1.07×10^−3^
Φ_KJ_	−2.61×10^−3^	−1.31×10^−4^
Φ_K_	4.89×10^−3^	−1.91×10^−6^
ϕ_J_	2.07×10^−7^	−3.62×10^−5^
ϕ_JK_	−1.74×10^−4^	−7.25×10^−5^
ϕ_K_	−1.51×10^−2^	3.75×10^−3^
*χ*_aa_ (Cl1)	−50.00	27.60
*χ*_bb_ (Cl1)	15.91	−62.08
*χ*_cc_ (Cl1)	34.10	34.49
*χ*_ab_ (Cl1)	−38.41	27.34
*χ*_ac_ (Cl1)	0.00	−0.57
*χ*_bc_ (Cl1)	0.00	1.70
*χ*_aa_ (Cl4)	−58.30	−31.02
*χ*_bb_ (Cl4)	21.58	13.34
*χ*_cc_ (Cl4)	36.71	17.685
*χ*_ab_ (Cl4)	−34.93	−38.78
*χ*_ac_ (Cl4)	0.00	−34.29
*χ*_bc_ (Cl4)	0.00	−21.03
*μ*_a_	−0.23	0.51
*μ*_b_	−1.88	−0.67
*μ*_c_	0.00	−1.41

^[a]^ Equilibrium and ground state rotational constants in MHz; quartic centrifugal distortion constants in kHz; sextic centrifugal distortion constants in Hz; nuclear quadrupolar coupling constants in MHz; ground state dipole moment components in D.

### Vibrational Spectrum of HCFC‐132 b Conformers

This subsection is devoted to the elucidation of the HCFC‐132b normal modes of vibration and their experimental characterization driven by theoretical simulations. A complete analysis of the absorption features involving combination‐, overtone‐ and hot‐transitions, as well as the understanding of the corresponding vibrational dynamics associated to anharmonic couplings is out the present scope and it will be deferred to a subsequent work extending the analysis beyond 3500 cm^−1^ and considering potential absorptions from the minor isotopologues containing ^37^Cl. The HCFC‐132b molecule possesses eighteen normal modes of vibration which can be classified according to the symmetry properties of the equilibrium configuration of the two conformers that, from the spectroscopic point of view, behave as two distinct species. The most stable rotamer, belonging to the *C_S_* symmetry point group, is an asymmetric nearly prolate rotor with an asymmetry parameter *κ*=−0.96. The *a* and *b* principal axis of inertia define the symmetry plane, with the *c* axis perpendicular to it. The eighteen normal modes of vibration, therefore, can be classified as 11 *A*’ ⊕ 7 *A”*, with *A*’ vibrations giving rise to hybrid *a*/*b* bands, and vibrations of *A*” symmetry producing *c*‐type absorptions. The rotamer of *C*_1_ symmetry, whose principal axis of inertia are depicted in Figure 1, has an asymmetry parameter *κ*=−0.70. Clearly, all vibrational normal modes belong to the *A* irreducible representation and therefore they can produce hybrid *a*/*b*/*c* bands with the magnitude of the various components depending on the orientation of the transition dipole moment. Transition frequencies and IR intensities computed according to the ChS within the double‐harmonic approximation are listed in Table 3 for both conformers. Harmonic intensities within the ChS, $I_{i}^{ChS}$ of each normal mode *i* have been obtaining according to the following equation:^[16]^

(1) $$I_{i}^{ChS}=I_{i}^{CCSD(T)/VTZ}+\Delta I_{i}^{MP2/(T-Q)}+\Delta I_{i}^{MP2/CV}$$

**Table 3:** Harmonic frequencies (cm^−1^) and IR intensities (km mol^−1^) of the vibrational normal modes of HCFC‐132b conformers.

	Conf. 1 (*C_S_*)		Conf. 2 (*C*_1_)	
	***ω*** ^***ChS***^	$I_{harm}^{ChS}$	***ω*** ^***ChS***^	$I_{harm}^{ChS}$
*ω*_1_	3121	7.17	3183	0.39
*ω*_2_	1472	10.58	3109	7.21
*ω*_3_	1333	22.47	1474	9.59
*ω*_4_	1253	84.00	1335	6.95
*ω*_5_	992	201.08	1269	85.92
*ω*_6_	803	11.68	1216	74.52
*ω*_7_	782	101.74	1136	176.01
*ω*_8_	561	12.78	1052	151.81
*ω*_9_	439	0.19	908	28.92
*ω*_10_	284	1.10	839	36.21
*ω*_11_	172	0.63	677	35.13
*ω*_12_	3192	0.77	582	18.03
*ω*_13_	1287	72.59	444	0.02
*ω*_14_	1120	92.99	427	1.70
*ω*_15_	919	9.33	329	0.15
*ω*_16_	418	0.06	314	2.08
*ω*_17_	329	0.88	172	0.86
*ω*_18_	105	2.29	100	1.83

where the first term on the r.h.s. is the harmonic intensity at the CCSD(T)/cc‐pVTZ level, while the second and the third terms account for the enlargement of the basis set and the contribution from the correlation of core electrons, respectively. The former is obtained as the difference between MP2 values computed with the cc‐pVQZ and cc‐pVTZ basis sets, while the latter is the difference between intensities calculated at the MP2/cc‐pCVTZ level by correlating all and only valence electrons. While representing an empirical relation, the reliability of this approach has been shown to provide accurate predictions for different halogenated molecules.[^12^, ^13^, ^16^]

**Figure 3:**
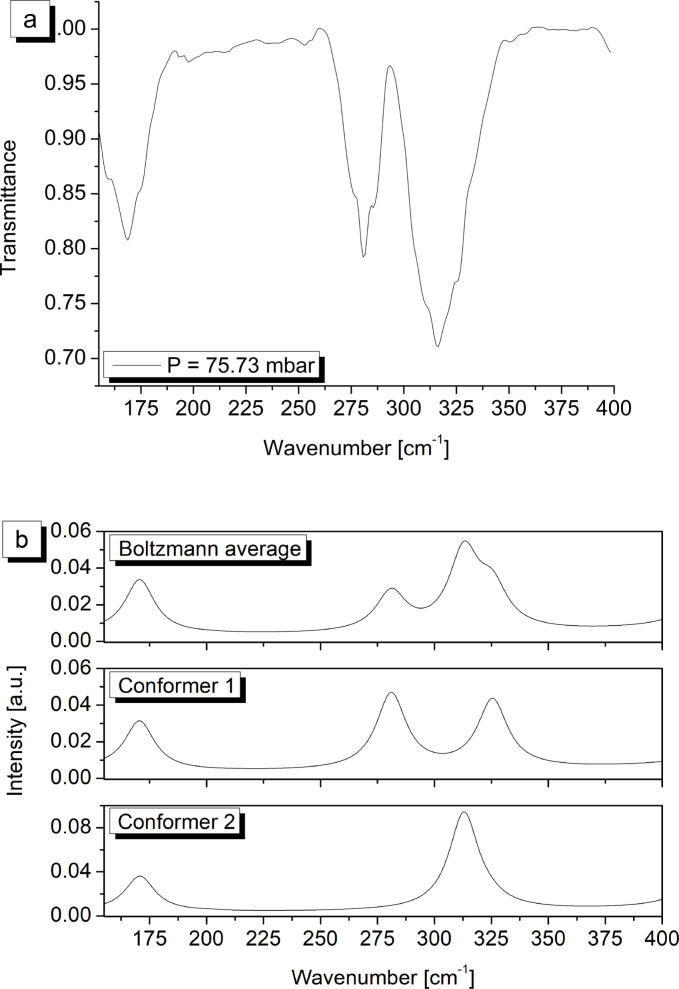
(a) HCFC‐132b experimental FTIR spectrum between 155 and 400 cm^−1^ (T=295.2±1.1 K, resolution=2.0 cm^−1^, optical path length=200 mm). (b) HCFC‐132b simulated spectrum: overall spectrum due to both conformes (top panel) and separate contributions from conformer 1 (middle) and conformer 2 (bottom).

**Figure 4:**
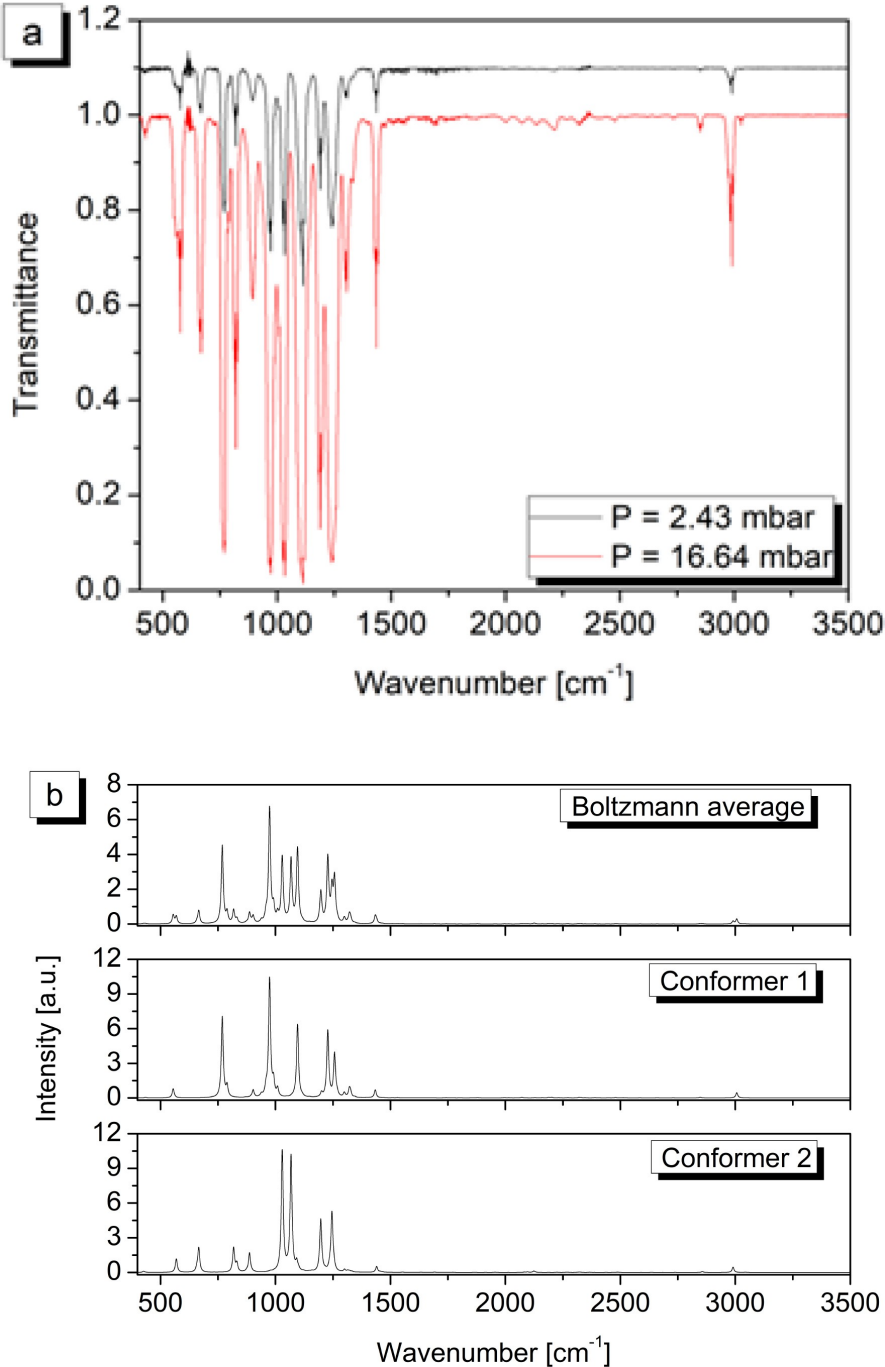
(a) HCFC‐132b experimental FTIR spectrum between 400 and 3500 cm^−1^ (T=295.2±1.1 K, resolution=0.5 cm^−1^, optical path length=134 mm). (b) HCFC‐132b simulated spectrum: overall spectrum due to both conformes (top panel) and separate contributions from conformer 1 (middle) and conformer 2 (bottom).

The FTIR spectra in the 150–400 cm^−1^ and 400–3500 cm^−1^ spectral intervals are reported in Figure 3 and 4 respectively, where they are also compared with the computed spectra of both conformers. In addition, the same figures also show the overall simulated spectrum resulting from the Boltzmann average of the two synthetic traces. Transition frequencies experimentally measured for both conformers are collected in Table 4 together with the corresponding counterparts and anharmonic IR intensities computed according to the ChS:DSD hybrid force field obtained by mixing ChS harmonic frequencies with anharmonic contributions evaluated at the DSDPBEP86/jun‐cc‐pV(T+*d*)Z level of theory.

**Table 4:** Fundamental vibrational frequencies (cm^−1^) and anharmonic intensities (km mol^−1^) of the normal modes of HCFC‐132b conformers.

	Conf. 1 (*C_S_*)			Conf. 2 (*C*_1_)		
	***ν*** ^***Exp*** **.[*a*]**^	***ν*** ^***ChS***^	$I_{anh}^{ChS}$	***ν*** ^***Exp*** **.[*a*]**^	***ν*** ^***ChS***^	$I_{anh}^{ChS}$
*ν*_1_	2990.1	2988	6.84	3030.6	3042	0.51
*ν*_2_	1434.5	1434	10.26	2985.0	2994	7.39
*ν*_3_	1302.4	1299	14.81	n.a.^[*b*]^	1435	6.61
*ν*_4_	1234.2	1227	70.83	1305(1)	1304	6.94
*ν*_5_	972.8	974	129.59	1244.0	1242	78.27
*ν*_6_	786.2(5)	790	4.84	1189.1	1186	58.05
*ν*_7_	768.4	769	107.48	1114.3	1110	161.31
*ν*_8_	555.0	555	12.28	1030.7(5)	1032	144.30
*ν*_9_	434.9	435	0.32	894.7	893	26.14
*ν*_10_	281(1)	282	1.02	820.2	822	30.56
*ν*_11_	168(1)	171	0.63	667.4	668	32.99
*ν*_12_	n.a.	3052	0.44	576.0	576	17.95
*ν*_13_	1258(1)	1257	53.80	n.a.	440	0.10
*ν*_14_	1100.4	1096	80.47	423.8	424	1.44
*ν*_15_	903.9	903	9.30	n.a.	325	0.14
*ν*_16_	n.a.	417	0.03	315(1)	312	2.15
*ν*_17_	327(1)	327	0.90	168(1)	170	0.75
*ν*_18_	^[*c*]^	105	2.26	^[*c*]^	97	1.86

^[*a*]^ Figures within parentheses are experimental uncertainties in the units of the last significant digit. The uncertainty amounts to 0.2 cm^−1^ if not reported. ^[*b*]^ Overlapped with *ν*_2_ of conformer 1. ^[*c*]^ Out of the acquired experimental spectral range.

The spectral region between 150 and 400 cm^−1^ features the presence of three main absorptions located respectively at 168 cm^−1^, 281 cm^−1^ and 315 cm^−1^, clearly observable in Figure 3. According to the quantum chemical calculations, the *C_S_* conformer is expected to have three fundamental vibrations coherent with these observations: *ν*_11_ at 171 cm^−1^, *ν*_10_ at 282 cm^−1^ and *ν*_17_ at 327 cm^−1^. The first one is predicted with an intensity of 0.63 km mol^−1^ which however overlaps with the *ν*_17_ fundamental of the *C*_1_ conformer having a strength of 0.75 km mol^−1^ and resonating at 170 cm^−1^. Therefore, both the normal modes appear as the carriers of the observed band. The absorption feature, measured at 281 cm^−1^, can be safely assigned to the *ν*_10_ band of the most stable rotamer which occurs with an intensity of 1.02 km mol^−1^ according to the ChS:DSD hybrid computations. The last observed band might, at first glance, be linked to the *ν*_17_ fundamental of the *C_S_* conformer, whose intensity at ChS:DSD level is 0.90 km mol^−1^. However, the simulations carried out for the *C*_1_ rotamer indicate that this band is rather due to the *ν*_16_ of the latter species, which gives rise to a much stronger absorption predicted at 312 cm^−1^ with an intensity of 2.15 km mol^−1^ (recall that, anyway, it has to be scaled for the relative population of this species). Finally, inspection of the spectrum reveals the presence of a shoulder discernible at 327 cm^−1^ that can be assigned to normal mode *ν*_17_ of the main rotamer. Moving to the region above 400 cm^−1^, the weak signal with absorption maximum at 423.8 cm^−1^ can be assigned to the *ν*_14_ band of the *C*_1_ conformer (1.4 km mol^−1^); the shoulder at about 435 cm^−1^ well correlates with the *ν*_9_ transition of the main rotamer which is predicted at 435 cm^−1^ with an intensity of 0.32 km mol^−1^. Still with reference to this rotamer, the *ν*_8_ band can be observed at 555.0 cm^−1^, whereas the absorptions at 576.0 cm^−1^ and 667.4 cm^−1^, which initially could not be assigned by considering only this species, do originate from the *ν*_12_ and *ν*_11_ fundamental transitions, respectively, of the *C*_1_ rotamer, which are calculated to occur at 576 cm^−1^ (*I*=17.9 km mol^−1^) and 668 cm^−1^ (*I*=32.9 km mol^−1^). Moving on within the atmospheric spectral window, the *ν*_10_, *ν*_9_ and *ν*_8_ fundamental transitions of the *C*_1_ rotamer have been identified, driven by quantum chemical calculations, with the medium‐strong features located at 820.2 cm^−1^, 894.7 cm^−1^ and 1030.7 cm^−1^, respectively. This spectral region also bears the absorptions arising from the *ν*_7_ (768.2 cm^−1^), *ν*_6_ (786.2 cm^−1^), *ν*_15_ (903.9 cm^−1^) and *ν*_5_ (972.8 cm^−1^) fundamentals of the *C_S_* conformer as well as a number of combination and hot bands whose detailed assignment will be postponed to a subsequent work. The spectral interval between 1060 and 1160 cm^−1^ is dominated by a very strong absorption due to a bundle of bands among which the *ν*_14_, at 1100.4 cm^−1^, and the *ν*_7_, at 1114.3 cm^−1^, fundamentals of *C_S_* and *C*_1_ rotamers, respectively, can be picked out. These observations are in good agreement with ChS:DSD computations that place these transitions at 1096 cm^−1^ and 1110 cm^−1^ with intensities of about 80.5 and 161.3 km mol^−1^, respectively. The last portion of the atmospheric window is also characterized by a number of strong absorptions among which the most important one is probably the *ν*_6_ band of the *C*_1_ conformer at 1189.1 cm^−1^. Furthermore, the *ν*_4_ and *ν*_13_ fundamental transitions belonging to the main conformer assigned at 1234.2 and 1258 cm^−1^, respectively, coalesce with the *ν*_5_ fundamental (1244.0 cm^−1^) of the second rotamer producing a broad feature spanning over the 1210 cm^−1^ to 1280 cm^−1^ range. This is immediately followed by the *ν*_3_ fundamental of the main conformer, observed at 1302.4 cm^−1^ and predicted at 1299 cm^−1^, whose R‐branch overlaps with the *ν*_4_ band of the *C*_1_ rotamer, which has been measured at 1305 cm^−1^ again in good agreement with ChS:DSD theoretical predictions (1304 cm^−1^). To conclude with the identification of the fundamental vibrations, the *ν*_2_ normal mode of the *C_S_* conformer gives rise to an a/b‐hybrid band, with prevalence of a‐type character, located at 1434.5 cm^−1^, in excellent agreement with theoretical predictions (1434 cm^−1^) and which obscures the nearby *ν*_3_ band of the *C*_1_ rotamer expected at about 1435 cm^−1^ with an intensity around 7 km mol^−1^ which lowers to 3 km mol^−1^ taking into account the relative abundance of the minor species. Finally, the region between 2940 and 3040 cm^−1^ hosts the highest‐frequency fundamental vibrations: the *ν*_1_ normal mode of the main rotamer produces a well defined band at 2990.1 cm^−1^, whose *P*‐branch overlaps with the *ν*_2_ vibration of the *C*_1_ confomer, with maximum measured at 2985.0 cm^−1^. The *ν*_1_ normal mode of the same rotamer produces a weak band, in accordance with ChS:DSD computations, at 3030.6 cm^−1^ whereas no absorptions coming from the *ν*_12_ vibration of the *C_S_* species have been observed, probably due to the low intensity of this transition.

### Radiative Efficiency and Global Warming Potential

In order to determine the RE of HCFC‐132b, its photo‐absorption cross section spectrum has been first derived by means of least squares analysis of multiple absorption spectra according to the following procedure.[^26^, ^27^] For each spectrum measured at varying pressure, the point‐by‐point absorbance cross section per molecule (cm^2^ molecule^−1^) of the radiating species, $\sigma (\tilde{\nu })$ , has been calculated from the measured infrared absorbance according to

(2) $$\sigma \left(\tilde{\nu }\right)=\frac{ln10A(\tilde{\nu })}{N_{A}cl}$$

where $A(\tilde{\nu })$ is the absorbance at wavenumber $\tilde{\nu }$ , *l* (cm) is the optical path‐length, *c* is the sample concentration (mol cm^−3^), and *N_A_* is Avogadro's number. After checking the linearity of the Beer‐Lambert's law at each wavenumber in the whole concentration range adopted during the experiments (Beer's law plots are provided in Figures S.1 and S.2 of the supporting information), the final photo‐absorption cross section spectrum at each wavenumber has resulted from the average of the absorbance cross section obtained at the each different HCFC‐132b pressures employed.

REs have calculated based on the Pinnock's narrow band model (NBM)^[28]^ using Equation 3:

(3) $$RE=\sum_{n=1}^{N}\left[\int_{{\tilde{\nu }}_{i,1}}^{{\tilde{\nu }}_{i,2}}\sigma \left(\tilde{\nu }\right)d\tilde{\nu }\right]F_{\sigma }^{i}$$

where $\sigma (\tilde{\nu })$ is absorbance cross section as defined in Eq. 2, integrated over the spectral range between ${\tilde{\nu }}_{i,1}$ and ${\tilde{\nu }}_{i,2}$ and $F_{\sigma }^{i}$ is the radiative forcing per unit cross section of the global‐annual mean atmosphere (GAM). In particular, the RF of the GAM worked out by Shine and Myre,^[29]^ which includes molecule dependent adjustments for stratospheric temperature, has been used. Besides considering experimental cross section spectra, the HCFC‐132b RE has been also derived based on the computed quantm chemical counterpart that, however, neglects the underlying ro‐vibrational structure. Following the procedure recently validated,^[30]^ computed stick spectra have been convoluted with a Lorentzian function with a half‐width at half‐maximum (HWHM) of 30 cm^−1^ in order to effectively consider the spectral bandshape. The convoluted spectrum has been subsequently used for the calculation of the anharmonic integrated absorption cross section over the series of integration intervals required by the NBM, Eq. 3. In all cases, the RE evaluation has been conducted in the range from 155 cm^−1^ to 3000 cm^−1^.

The model developed by Pinnock assumes a well‐mixed distribution of gases across altitude and latitude. However, it has been demonstrated that when such conditions are not met, a correction term for the gas lifetime (*τ*) must be introduced. In our study, we have adjusted the RE values by considering a *τ* of 3.5 years^[15]^ within the S‐shaped curve^[31]^ to account for the non‐uniform vertical profile and horizontal distribution, assuming that OH degradation is the dominant removal process for this compound. The calculation of REs was performed using a home‐made software. Further details about the methodology, are reported in Ref. [30].

**Figure 5:**
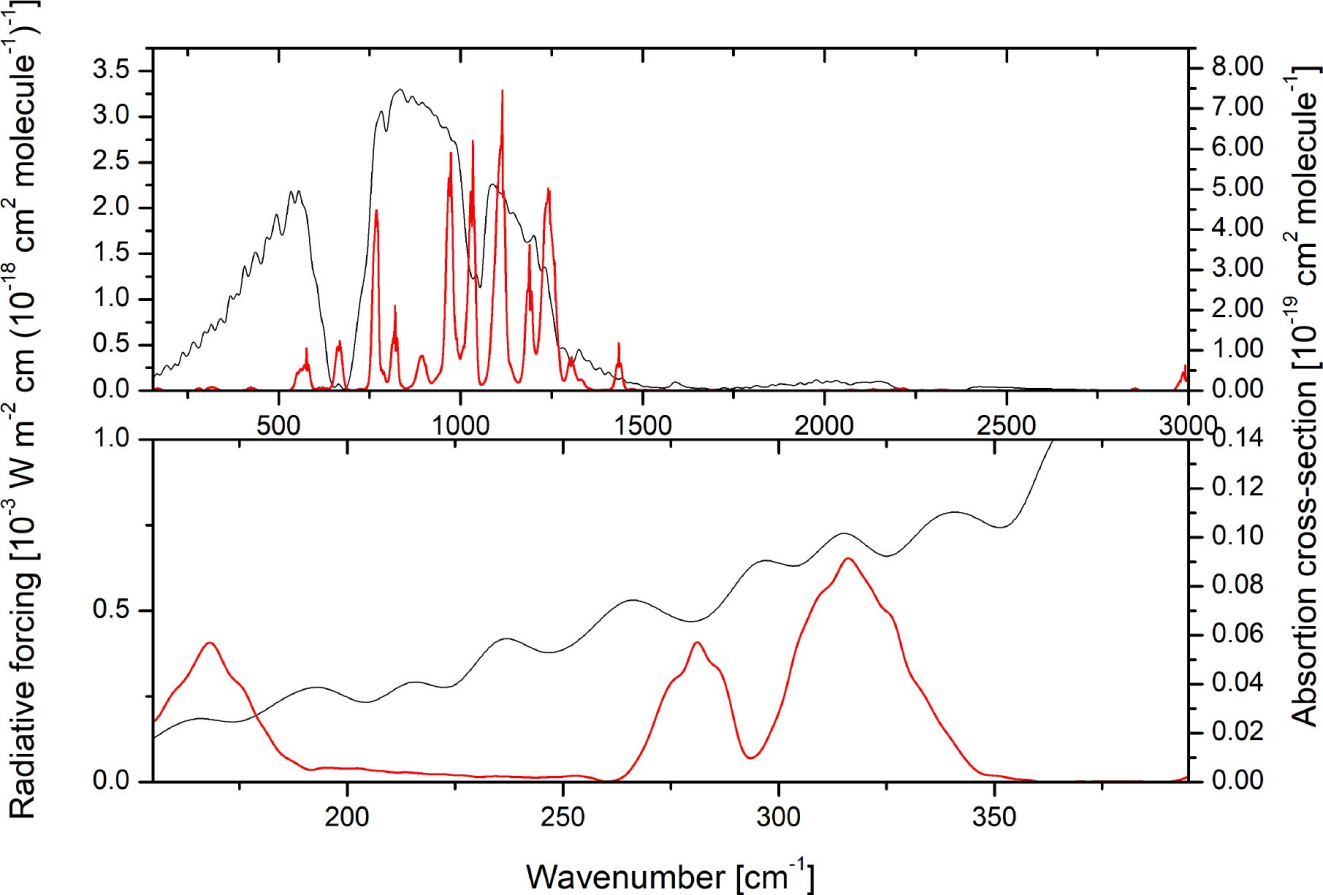
IR absorption cross section spectrum of HCFC‐132b (red line) superimposed to the RF per unit cross section of the GAM (black solid line): (top) whole spectral range and (bottom) magnified view in the region between 155 and 375 cm^−1^.

The absorption cross‐section provides a measure of the overall strength of IR absorption and its variation with wavenumber. While the atmospheric lifetimes of HCFCs are generally shorter than those of the analog CFCs, similar to the latter ones, most of the long‐wave absorption of HCFC‐132b occurs in the atmospheric window region between 800 and 1200 cm^−1^, as shown in Figure 5a, which reports the photo‐absorption cross section spectrum experimentally retrieved in this work over the 155–3000 cm^−1^ range superimposed to the RF of the GAM. In passing, it should be noted that the total integrated IR absorption cross section over this spectral range amounts to 10.1×10^−17^ cm molecule^−1^, of which 6.0×10^−17^ cm molecule^−1^ fall within the atmospheric window. The uncertainty of the measured absorption cross section spectrum has been estimated to be 2 % over the 400–3500 cm^−1^ and about 13 % for the 150–400 cm^−1^ region. The RE and ERE retrieved from the measured photo‐absorption spectrum are reported in Table 5. Let us recall that EREs are better predictors of surface temperature response than instantaneous REs.^[29]^ The same table also collects the theoretical REs obtained from the Boltzmann average of the absorption cross section spectra quantum chemically computed for the *C_S_* and *C*_1_ rotamers. Table 5 also reports the values listed in the last WMO ozone depletion assessment report.^[15]^ As evident, the experimentally derived RE and ERE are significantly lower (by 14 %) than those collected in the WMO report, whereas an excellent agreement between experimental and quantum chemical values is noted. An ERE of HCFC‐132b obtained from anharmonic quantum chemical calculations, that well matches with the present experimental determination, has already been reported previously,^[30]^ however in in this work, we have improved these theoretical ERE values by including the second conformer. The 13 % overestimation in the RE taken from the WMO 2022 ozone depletion assessment is not surprising, being based on theoretical spectra computed within the double‐harmonic approximation^[32]^ which totally neglects contributions from overtone‐ and combination‐ transitions, intensity redistribution due to anharmonic coupling and it generally overestimates fundamental absorption cross sections. Thus, while computationally simpler and less demanding, resorting to the double‐harmonic treatment of vibrations can lead to significant differences compared to experimental results, with apparently accurate outcomes stemming from error compensation.

**Table 5:** Experimental and theoretical radiative efficiency (W m^−2^ ppbv^−1^) of HCFC‐132b.

	Exp.	Theo.	WMO^[15]^
RE	0.187	0.194	0.214
ERE	0.168	0.174	0.192

Furthermore, in this work, we also measured absorption contributions between 150 and 500 cm^−1^ which are magnified in Figure 5b, a range that is usually not considered in experimental determinations^[33]^ of REs. The HCFC‐132b RE due to the low‐frequency absorptions has been quantified to be 0.3 % of the overall forcing.

**Figure 6:**
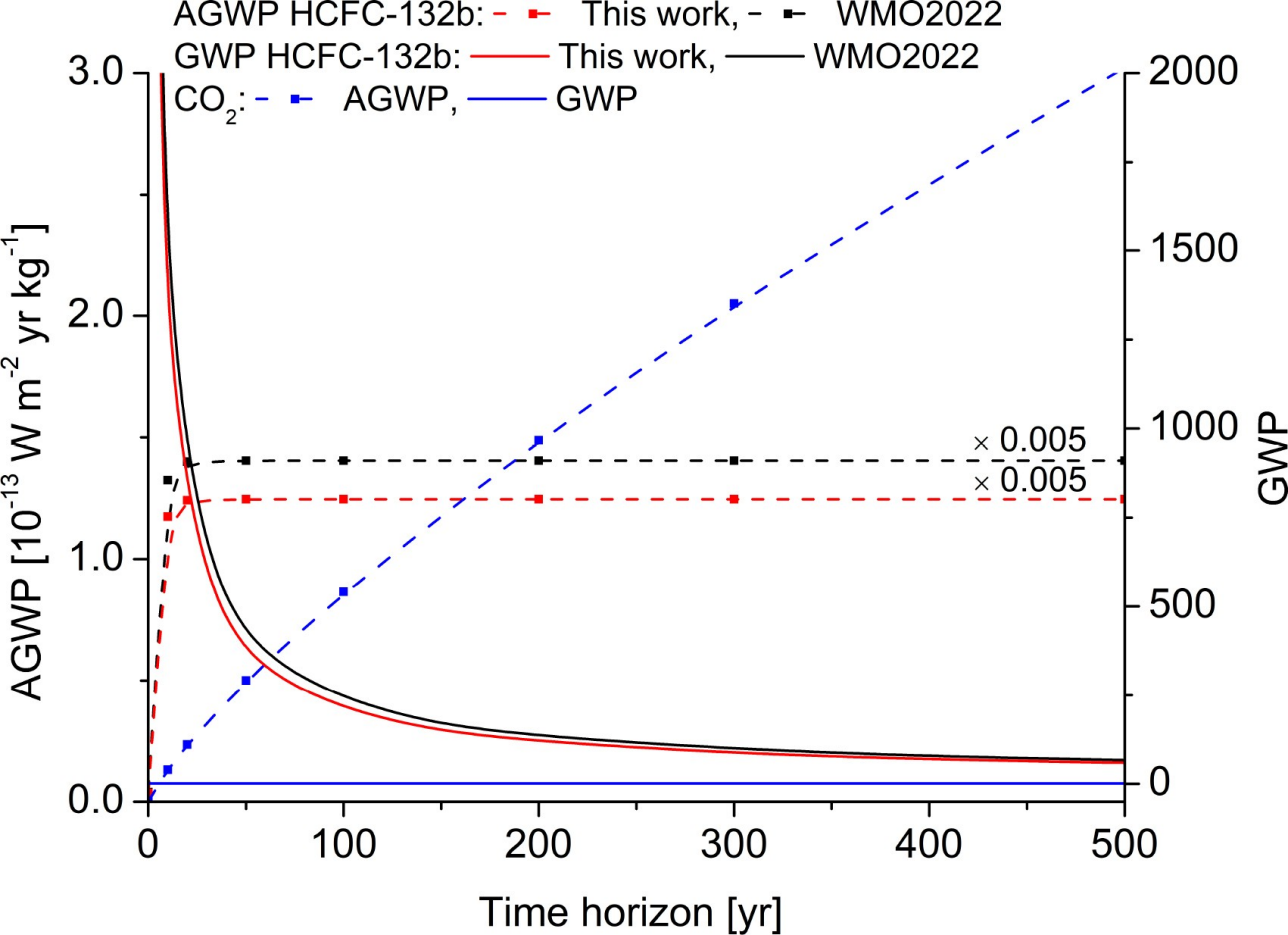
Absolute Global Warming Potential (AGWP) and Global Warming Potential (GWP) of HCFC‐132b determined in this work (red line) and in the WMO 2022 report on ozone depletion (black line). AGWP and GWP of CO_2_ are also reported (blue line).

The global warming potential (GWP) of HCFC‐132b has been derived from the experimental ERE here determined and the atmospheric lifetime of 3.5 yr suggested in the WMO 2022 report^[15]^ according to:

(4) $$GWP_{HCFC-132b}\left(\tau_{H}\right)=\frac{AGWP_{HCFC-132b}\left(\tau_{H}\right)}{AGWP_{CO_{2}}\left(\tau_{H}\right)}$$

where *τ_H_* denotes the time horizon over which the global warming potential is evaluated and *AGWP_i_* is the absolute global warming potential of the species *i*. The approach used to generate radiative metrics provided here is based on the 2019 CO_2_ abundance and is fully described in Hodnebrog et al..^[31]^ The CO_2_ AGWPs for the 20‐, 100‐, and 500‐year time horizons are 2.434×10^−14^, 8.947×10^−14^, and 3.138×10^−13^ W m^−2^ yr kg^−1^, respectively, which are consistent with the values stated in IPCC AR6.^[15]^ The GWP values for HCFC‐132b have been estimated as 1006, 275, and 78 for the 20, 100, and 500‐year time horizons, respectively. The values reported by the WMO in 2022 are 1190, 332, and 95^[15]^ which are higher than the present results by about 15 %, as a consequence of the aforementioned overestimation on the RE. As shown in figure 6, even if the AGWP remains constant across different time horizons, the most significant differences in GWP are observed in the first 20 years. This is due to the short lifetime of the molecule, governed by natural atmospheric removal processes that prevent indefinite increases in its atmospheric concentration, even with stable emission rates.^[34]^ Consequently, its strong warming influence diminishes rapidly over a few decades. It is important to note that treating long‐ and short‐lived climate pollutants in the same manner fails to capture their contrasting dynamics accurately.[^34^, ^35^] This highlights the importance of refining RE values and enhancing the accuracy of spectroscopic properties of molecules to ensure reliable climate metrics.

## Conclusions

Among anthropogenic atmospheric gases, HCFCs, originally introduced as *ad interim* replacements of CFCs, can seriously contribute to global warming mainly because of their strong IR absorptions in the 8–12 *μ*m atmospheric windows. Despite the existing regulations, HCFC‐132b has been unexpectedly detected in the atmosphere with increasing concentrations. As this molecule has no known end‐uses, it is not surprising that its spectroscopic properties, which are of relevance for both atmospheric monitoring and for the determination of climate metrics, escaped any dedicated investigation. In the present work, the rotational‐ and vibrational spectroscopic properties of HCFC‐132b have been characterized for the first time by adopting an integrated experimental and theoretical approach with the final aim of determining its radiative efficiency and global warming potential. The interplay between theory and experiment pointed out that the observed spectroscopic behaviour is actually due to the the mixture of two different rotamers, having *C_S_* and *C*_1_ symmetry respectively, which stem from the rotational about the C−C bond and are only 0.4 kcal mol^−1^ apart. More in detail, the equilibrium molecular structure of the two rotamers has been computed by the ChS CCSD(T)‐based composite scheme, which is expected to deliver and accuracy of 0.2 mÅ and 0.2° for bond lengths and angles, respectively. From the equilibrium structures, ground state rotational constants have been derived and supplemented through centrifugal distortion parameters, nuclear quadrupole coupling constants and dipole moment components, thus allowing the simulation of the pure rotational spectra. Then, IR transition frequencies and intensities beyond the double‐harmonic approximation have been predicted by using an hybrid force field, in which ChS harmonic properties have been mixed with anharmonic contributions evaluated using the DSDPBEP86 density functional. These quantum chemical predictions have been used to drive the interpretation, in the fundamental band regions, of the FTIR spectrum recorded experimentally over the 150–3500 cm^−1^ range. The observed signals have been found to be completely coherent with the Boltzmann average of the IR spectra simulated for the two conformers. Finally, the absorption cross section spectrum of HCFC‐132b has been experimentally derived over the spectral range between 155 and 3000 cm^−1^, which has been employed to obtain the first experimental measurement of the effective radiative efficiency of this molecule, which has resulted to be 0.168 W m^−2^ ppbv^−1^ by considering both stratospheric and lifetime adjustments. Therefore, the HCFC‐132b RE listed in the WMO 2022 report on ozone depletion is overestimated by 13 %, mainly because it is based on a theoretical estimate of the absorption cross‐section spectrum obtained within the double‐harmonic approximation. Indeed, the RE here experimentally derived is in excellent agreement with the present quantum mechanically computed value (0.174 W m^−2^ ppbv^−1^), obtained by adopting a quantum chemical workflow featuring non‐empirical calculation of anharmonicity in both transition frequencies and intensities, inclusion of low‐frequency contributions and consideration of conformational distribution^[30]^ (a comparison between experimental and quantum chemical IR absorption cross section spectra is given in Figure S.3 of the SI). From the retrieved RE, the GWP of HCFC‐132b has been estimated over the 20‐, 100‐ and 500‐year time horizons (1006, 275, and 78, respectively), showing that WMO reported values are overestimated by about 15 % as a consequence of the overestimation in the REs. All in all, besides providing the first rotational/vibrational spectroscopic characterization of this molecule and the first experimental assessment of its RE and GWP, our analysis demonstrates that, in addition to atmospheric lifetimes, variations in REs can significantly affect GWP calculations. It is crucial that these values are continuously updated and strive for the highest accuracy. These metrics can influence both private and public decision‐making processes, as they quantify the carbon budget and guide mitigation policies. They also serve as the framework for predicting future global surface temperature changes under specific emissions scenarios, thereby informing adaptation guidelines to climate change.

## Methods

### Computational

The molecular structure and vibrational‐rotational spectroscopic properties of HCFC‐132b were predicted by quantum chemical computations carried out at different levels of theory in order to properly treat both electronic and nuclear problems. The equilibrium structure was computed by exploiting the cheap composite scheme (ChS)^[36]^ that is based on the coupled cluster theory with singles, doubles and a perturbative estimate of triples, CCSD(T),^[37]^ and on top of it applies extrapolation to the complete basis set (CBS) limit and core‐correlation effects. The ChS was also used for computing harmonic frequencies of vibration, quartic centrifugal distortion constants and the nuclear quadrupolar coupling constants due to the presence of the Cl nucleus.^[38]^ More in detail, within the ChS the estimate of the target property *p* ^*ChS*^ (*p* standing for structural parameters, quartic centrifugal distortion constants, nuclear quadrupolar coupling constants or harmonic vibrational frequencies) is obtained by adding to the value computed at CCSD(T) level in conjunction with the cc‐pVTZ basis set corrections that accounts for the CBS extrapolation and core‐valence correlation evaluated using the second‐order Møller‐Plesset (MP2)^[39]^ perturbation theory. The CBS limit is estimated on the basis of the *n* ^−3^ equation applied with cc‐VTZ and cc‐pVQZ basis sets, while the core‐valence contribution is evaluated as the difference between the values computed at the MP2 level in conjunction with the cc‐pCVTZ basis set by correlating all electrons and within the frozen core approaximation, respectively. Vibrational anharmonic contributions to the computed harmonic properties were evaluated resorting to density functional theory (DFT). According to the recent literature, double‐hybrid functionals like B2PLYP^[40]^ and (rev‐)DSDPBEP86^[41]^ joined with a triple‐*ζ* basis set can be recommended for the purpose in view of their good performance in the prediction of structural and ro‐vibrational spectroscopic properties.[^17^, ^19^, ^42^, ^43^] In the present work, the DSDPBEP86 functional in conjunction with the jun‐cc‐pVTZ basis set^[44]^ was employed as it has been demonstrated to perform very accurately when applied to the evaluation of the RE of halogenated hydrocarbons.[^12^, ^13^] The basis set was supplemented by an additional set of *d* functions on the Cl atom in order to improve the accuracy of the results.[^19^, ^45^] At all the levels of theory considered, geometry optimizations were first carried out, followed by evaluation of analytical Hessians. Cubic and semidiagonal quartic force constants and second‐ and third‐order derivatives of the dipole moments were obtained through numerical differentiation of analytical Hessian matrices, and first‐order derivatives of the dipole moment surface, respectively. The relevant spectroscopic parameters were derived in the framework of vibrational perturbation theory to second‐order (VPT2)[^46^, ^47^, ^48^] by using the computed equilibrium geometries, harmonic properties and anharmonic force constants. Coupled cluster computations were performed by using the CFOUR software,^[49]^ while MP2 and DFT calculations were carried out employing the Gaussian16 suite of programs^[50]^ which was also adopted for applying VPT2 through its built‐in generalized VPT2 engine.[^51^, ^52^] On the basis of the available literature, the applied computational methodology is expected to provide equilibrium geometries accurate within 2 mÅ for bond lengths and 0.1‐0.2° for bond angles, and fundamental transition frequencies and intensities predicted with an average error around 5 cm^−1^ and a few km mol^−1^, respectively.[^24^, ^53^, ^54^, ^55^, ^56^]

### Experimental

The gas‐phase IR spectra of HCFC‐132b were recorded in the range of 150–3500 cm^−1^ by employing a Bruker Vertex 70 FTIR instrument. More in detail, spectra in the 150–400 cm^−1^ region were acquired at a resolution of 2 cm^−1^ using a 20.0 (±0.5) cm path length cell having polyethylene windows. For the 400–3500 range, the gas was introduced in a double walled, stainless steel cell, fitted with KBr windows and with an optical path‐length of 134.0 (±0.5) mm and the spectra were acquired at resolutions of 0.5 and 1.0 cm^−1^. For the vibrational analysis, the spectra were recorded at room‐temperature, 128 scans were averaged, and the pressure of the gas was varied in the range of 2.4–29.3 hPa. Determination of the absorption cross section spectra was performed according to a well consolidated experimental procedure.[^14^, ^56^, ^57^] For the measurements performed in the 400–3500 cm^−1^, spectra with a 0.5 cm^−1^ resolution were obtained at constant temperature (295.2±1.1 K) at nine different gas pressures in the range of 1.5–16.3 hPa and 128 interferograms were acquired for each spectrum in order to increase the signal‐to‐noise ratio. In the 150–400 cm^−1^ spectral region, absorption spectra were acquired at eleven different pressures, in the interval between 67.2–114.1 hPa, in the same experimental conditions described above. Pressure measurements were performed employing a capacitance vacuum gauge with a full‐scale range of 125 mbar and quoted manufacturer's full scale accuracy of 0.15 %. The gas cell was evacuated down to about 10^−2^ Pa before and after each sample and the background spectra were acquired. Adsorption of the gas sample on the cell walls was checked both by directly measuring the pressure, and by monitoring the absorption spectrum: it was found negligible over a period of 1 h, which is far longer than the typical time required to obtain a spectrum. The HCFC‐132b commercial sample was supplied by Ambinter with a stated purity of 95 %, and it was used without any further purification.

## Conflict of Interests

No conflict of interest to declare.

1

## Supporting information

As a service to our authors and readers, this journal provides supporting information supplied by the authors. Such materials are peer reviewed and may be re‐organized for online delivery, but are not copy‐edited or typeset. Technical support issues arising from supporting information (other than missing files) should be addressed to the authors.

Supporting Information

## Data Availability

The data that support the findings of this study are available from the corresponding author upon reasonable request.
