# Supplementary material for: The Radiative Efficiency and Global Warming Potential of HCFC‐132b
Source: Chemphyschem. 2024 Dec 4;26(1):e202400632. doi: 10.1002/cphc.202400632 (PMC11747578; doi:10.1002/cphc.202400632)
Supplement: Supplementary file 1 — Supporting Information [file CPHC-26-e202400632-s001.pdf]

ChemPhysChem

Supporting Information

The Radiative Efficiency and Global Warming Potential of HCFC-132b

Daniela Alvarado-Jiménez, Andrea Pietropolli Charmet, Paolo Stoppa, and Nicola Tasinato*

THE RADIATIVE EFFICIENCY AND GLOBAL WARMING POTENTIAL OF HCFC-132b

Daniela Alvarado-Jiménez,[a],[b] Andrea Pietropolli Charmet,[c] Paolo Stoppa,[c] Nicola Tasinato*[b]

Supporting Information

Figure S.1. Beer's plot of the integrated absorption cross section spectrum over the 400 - 3000 cm^{-1} range against the HCFC-132b pressure.
Figure S.2. Beer's plot of the integrated absorption cross section spectrum over the 155 - 400 cm^{-1} range against the HCFC-132b pressure.
Figure S.3. Comparison between the HCFC-132b absorption cross section spectrum determined experimentally over the 155 - 3000 cm^{-1} and computed at the DSDPBEP86-D3/jun-cc-pV(T+d)Z level of theory.

[a] D. Alvarado-Jiménez
IUSS Pavia

[b] D. Alvarado-Jiménez, Prof. Dr. N. Tasinato*
Scuola Normale Superiore
E-mail: nicola.tasinato@sns.it

[c] Prof. Dr. A. Pietropolli Charmet, Prof. Dr. P. Stoppa Dipartimento di Scienze Molecolari e Nanosistemi, Università Ca' Foscari Venezia

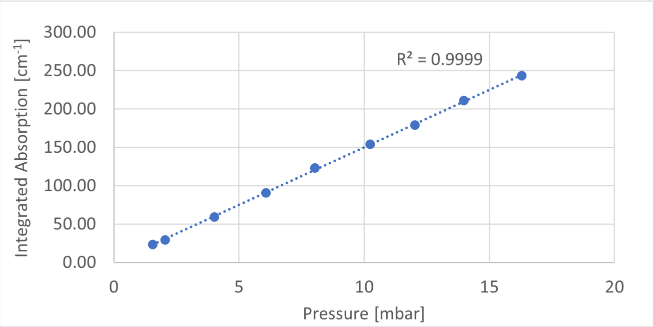

Figure S.1. Beer's plot of the integrated absorption cross section spectrum over the 400 - 3000 cm^{-1} range against the HCFC-132b pressure.

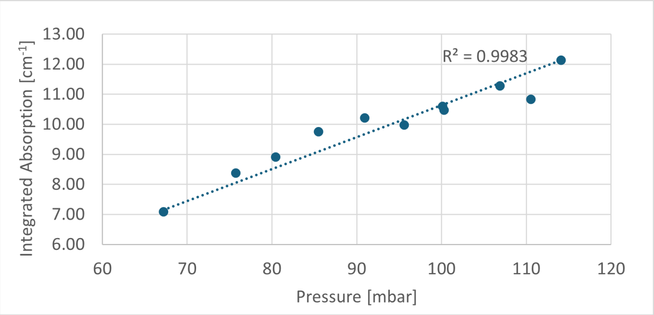

Figure S.2. Beer's plot of the integrated absorption cross section spectrum over the 155 - 400 cm^{-1} range against the HCFC-132b pressure.

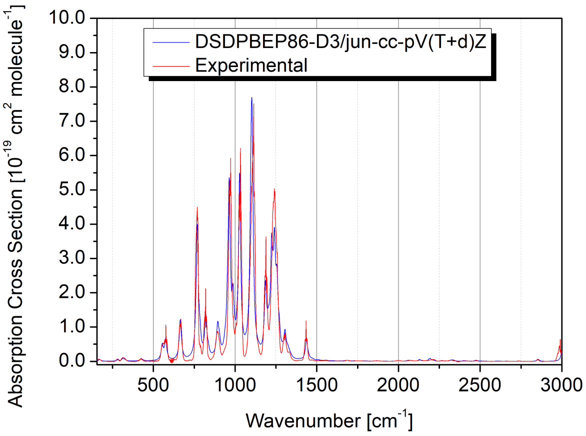

Figure S.3. Comparison between the HCFC-132b absorption cross section spectrum determined experimentally over the 155 - 3000 cm^{-1} and computed at the DSDPBEP86-D3/jun-cc-pV(T+d)Z level of theory.
